# A cross-sectional study of factors associated with regular dog walking and intention to walk the dog

**DOI:** 10.1186/s12889-022-12902-w

**Published:** 2022-03-22

**Authors:** Carri Westgarth, Robert M. Christley, Hayley E. Christian

**Affiliations:** 1 Department of Livestock and One Health, Institute of Infection, Veterinary and Ecological Sciences, Faculty of Health and Life Sciences, University of Liverpool, Leahurst Campus, Chester High Road, Neston, Cheshire, CH64 7TE UK; 2 Present Address: Dogs Trust, London, UK; 3 Telethon Kids Institute, The University of Western Australia, Crawley, WA Australia; 4 School of Population and Global Health, The University of Western Australia, Crawley, WA Australia

**Keywords:** Attitude, Dogs, Exercise, Intention, Pets, Public Health, Walking

## Abstract

**Background:**

Dog walking is important for public health and dog welfare, yet some owners do not walk with their dogs regularly. This study examined factors associated with participation in regular dog walking and intention to dog walk, in order to inform physical activity interventions.

**Methods:**

191 dog-owning adults from a UK community were surveyed about their participation in dog walking, intention to dog walk, attitudes and behavioural beliefs regarding dog walking, and dog and owner demographics. Principal components analysis identified owner profiles regarding attitudes and behavioural beliefs about dog walking. Univariable and multivariable logistic regression were used to identify factors associated with being a regular dog walker (achieving 150mins per week of dog walking) and having a high intention to dog walk (at least 30 mins per day for at least 5 days per week over the next month).

**Results:**

Participants walked with their dogs for a median 7 times/week and 230 total minutes/week; regular dog walkers 9 times/week (400 minutes/week), compared to twice/week for irregular dog walkers (45 minutes/week). Being a regular dog walker was positively associated with having a high level of intention to walk the dog in the next month (OR=12.1 95%CI=3.5-42.4, *P*<0.001), being married or living with a partner (OR=33.5, 95%CI=2.5-458.8, *P*=0.01), and higher scores on a dog walking habit index (OR=2.1, 95%CI=1.3-3.5, *P*<0.01). However, higher support from friends for walking was negatively associated with being a regular dog walker (OR=0.3, 95%CI=0.1-0.7, *P*<0.01). High intention to dog walk was associated with female owners (OR=4.7, 95%CI=1.2-18.5, *P*=0.03), dogs that lay on the sofa (OR=6.9, 95%CI=1.5-31.8, *P*=0.01), high levels of self-efficacy to walk the dog over the next month (OR=5.8, 95%CI=1.5-21.9, *P*=0.01), owner type with an attitude of high responsibility and enjoyment from walking (OR=2.1, 95%CI=1.2-3.8, *P*=0.02), and higher scores on a dog walking habit index (OR=1.9, 95%CI=1.0-3.7, *P*=0.05). Reporting someone else walks the dog was negatively associated with high intention (OR=0.1, 95%CI=0.0-0.7, *P*=0.02).

**Conclusions:**

Interventions to promote dog walking may benefit from increasing intention to dog walk in male owners, forming schedules and routines that involve multiple household members in dog walking, and establishing habits around dog walking. Interventions may also need to address how to overcome barriers and perceived challenges in regards to self-efficacy of dog walking, that may prevent intention from being translated into action.

## Background

Being physically active on a regular basis is a public health priority due to evidence of the many health benefits including reduced risk of chronic diseases such as heart disease, diabetes and cancers, and improvements to mental wellbeing [1, 2]. It is recommended that adults undertake a minimum of 150 minutes of physical activity per week but many struggle to achieve this, especially women, youth and those in higher income countries [3]. Even light physical activity, such as walking, is beneficial and the most significant reductions in risk occur for an uptake of low intensity activity [4]. Therefore interventions to promote walking are of interest to public health [5].

Dog walking is the most popular reason for visits to outdoor environments in England [6] and recent data suggests that up to 64% of UK dog owners achieve the recommended 150 minutes per week of physical activity through walking their dog [7], although some owners never or rarely walk with their dogs. Furthermore, dog walking rates appear to be lower in most other countries where research has been conducted such as Australia, North America and Taiwan [8, 9] except perhaps Japan [10]. A 2014 review highlighted that some types of owner-dog relationships, dogs and environments are more conducive to dog walking, and for some countries such as the UK, studies were scarce [11]. It is important to understand the barriers and motivators to participating in physical activity with a dog, so that interventions to promote human physical activity and improve canine health welfare can be effectively designed and targeted.

One previous study in a UK community has investigated factors associated with dog walking but only from the perspective of the dog’s welfare, in terms of factors associated with the dog being walked daily, by any of the household members [12]. Whilst not quite the same research question as factors associated with the owner’s involvement in dog walking (who may or may not be doing the dog walking), it showed that dogs that receive daily walks are: larger, more likely to lie on furniture and less likely to lie on laps, rarely growl at household members, rarely play chase games, live in single rather than multiple-dog households and in households with fewer human members, and the reason the dog was acquired was for a hobby [12].

More pertinent research in other countries has focused on owner’s activity with their dog(s), however only from the perspective of one household member (likely the person that does the most dog walking). For example, an Australian study found that owners are more likely to walk with their dog if: they feel that the dog provides motivation to walk more; if they feel that their dog provides social support for walking; if it is perceived the attitude of ‘significant’ others (e.g. family, other owners and veterinarian) toward daily dog walking (subjective norm) is positive; and, if they feel that dog-specific barriers to walking (e.g., my dog would be difficult to control, fear of other people’s dogs, difficulty in walking multiple dogs) are unlikely to discourage them from walking [13]. Likewise, dog owners from the same population who did walk with their dogs were more likely to be classed as ‘regular dog walkers’ (>90minutes per week) if they felt their dog provides motivation and/or social support for walking, and if they lived within 1.6km of a park with dog-supportive features [14]. The latter finding supports wider evidence that neighbourhood factors such as access to places suitable for walking are also associated with dog walking behaviour [15–17]. Numerous other studies also agree that the ‘attachment’ to the dog [18] and/or sense of ‘obligation’ [19–21] towards it are important correlates of dog walking behaviour in owners. Studies have also shown that psychosocial factors such as feelings of self-efficacy around dog walking predicts ‘stages of change’ of owners regarding their dog walking behaviour [19] and actual dog walking behaviour [21].

As the perceived motivation for walking and sense of obligation that a dog provides is clearly an important factor, the Australian dataset was also examined more deeply for factors associated with this perception that a dog provides motivation and encouragement for walking; positive associations include larger dogs, higher attachment to the dog, feeling that the dog enjoys going for a walk, believing that walking keeps dogs healthy, and high social support from family members for walking [22]. Conversely, the presence of children at home, and perceived dog-specific barriers to walking, are negatively associated with the perception that a dog provides motivation and encouragement for walking [22].

More recently, qualitative research in the UK into why owners walk their dogs suggests that perceiving owner mental health benefits and stress release from enjoying seeing their dog happy on a walk is important to owners [23]. This supports quantitative findings from Canada that intrinsic motivators (e.g. finding an activity pleasurable) seem to be more important with regard to dog walking behaviour than extrinsic motivators (for the purpose of a reward outside the activity itself, such as reducing feelings of guilt) [24]. Further, the establishment of dog walking as a habit or routine has recently been suggested to be important in both qualitative [23] and quantitative research [25]. In contrast, although social interaction with other people with others is a common outcome of owning pets, in particular through going for a dog walk [26–29], qualitative research suggests it is not felt to be a key motivating factor for dog walking for most owners or can even demotivate walking if the owner wants to avoid interacting with other people or dogs for various reasons [23, 30].

Based upon the previous limited research findings described, there are a number of key areas of investigation that future studies examining the correlates of dog walking in different population groups should address. These include demographic factors of both dogs and owners, measures of dog-owner interactions and relationship or ‘attachment’ strength, and the use of habits and routines for dog walking. In addition, studies should investigate owner perceptions and beliefs about dog walking, in particular the perceived benefits to be gained by both the dogs and owners from regular dog walking. This information would help to inform the content of future physical activity interventions for dog owners, both in the UK and worldwide. Given a specific lack of UK research into dog owners’ walking behaviour, it is important to discover what factors may be associated with differences in dog walking participation in UK dog owners in particular. Having an ‘intention’ to perform physical activity such as recreational walking is also a known to be strong predictor of the behaviour [31], including for dog walking [20], however factors associated with having this ‘intention to walk the dog’ has not been previously explored and should be investigated. As intention is a clear first step on the pathway to actualising behaviour, this information is important in order to understand how to motivate owners to make plans to take their dog for more walks, improving both canine welfare and human public health outcomes.

## Methods

### Aim

The first aim of this study was, for the first time, to investigate factors associated with owner dog walking in a UK sample, and more specifically in this case, participating in regular dog walking totalling at least 150 mins/week. The study also set out to investigate a much wider range of factors than has been previously examined elsewhere, including habit formation, perceived owner mental health benefits, and perception of dog activity needs regarding size and breed. A novel aspect of this study design was that all of the participants lived in the same geographic neighbourhood, thus had access to similar environmental opportunities for dog walking, effectively controlling for this influence. This study is also original as it investigates participation in dog walking by multiple household members, in order to reduce bias from survey participation by the household member who most often walks the dog, as likely in previous studies. Based on the preliminary research described above, we hypothesised that perceived owner health benefits from walking, in particular mental benefits, would be important, as would qualities of the dog-owner relationship around feelings of responsibility, and aspects of routine/habit-making in regards to dog walking. A second aim of this study was for the first time to investigate whether owner behavioural beliefs and attitudes towards dog walking could be used to identify common profile types of dog owners with similar perceptions, which may be useful for targeting interventions. Finally, as having a high intention to walk the dog was subsequently demonstrated to be a strong predictor of significant amounts of owner dog walking in this sample, and factors associated with having a high intention to walk the dog has never previously been examined, the third aim of this study was to also investigate this.

### Data collection design and setting

The data collection process and sample has been described previously [7, 32]. Briefly, a survey was conducted in a semi-rural area of 1280 households, by visiting all households up to a maximum of five times until an adult household member was contacted, and leaving surveys for each household member to complete and return by post or online. Separate surveys were also provided regarding each dog resident in the household. Questionnaires were returned for 191 dog-owning adults (aged 16 or above) from 113 households. The full surveys can be requested from the corresponding author.

### Outcome variables

#### Regular dog walking

Outcome items were based upon the validated RESIDE Neighbourhood Physical Activity Questionnaire (NPAQ) [33] and Dogs And Physical Activity (DAPA) Tool [34]. Of the 191 dog owners, 184 provided information on the frequency and minutes spent per week walking with their dog, jogging with their dog and, in addition, cycling with their dog. In previous testing, recall of frequency and duration of walking or jogging with the dog in the previous week has been shown to be reliable (ICC 0.98 and 0.94 respectively) [34], however, in the current study jogging and cycling were asked separately to walking. Walking, jogging or cycling with a dog were grouped together and termed ‘dog walking’.

Respondents were classified into ‘Regular Dog Walkers’ (RDW) if they walked (including jogged or cycled) with their dog for at least 150min/week total; or ‘Irregular Dog Walkers’ (IDW) who did not meet this criterion. Previous research has used 90min/week as the cut-off to describe regular (59%) versus irregular (41%) dog walkers and excluded those owners who did not walk with their dogs at all (n=146) [14]. Due to the higher minutes of dog walking observed in our study, and the smaller overall sample size, we chose 150 minutes per week, as this also aligns with the minimum physical activity recommendations for adults [35]. Some respondents volunteered further answers regarding time spent doing ‘other’ activities with their dog, such as training classes or ‘playing’ in the garden, but these activities were excluded from the primary outcome measure to reduce discrepancies between those who volunteered that information and those who did not report it. As previously reported, 18 dog owners reported no physical activity undertaken with their dog [7]; these were included in the categorisation of Irregular Dog Walkers.

#### Intention to dog walk

High intention to dog walk was adapted from previous ‘theory of planned behaviour’ based physical activity survey items [36], here measured as intention in the next month to walk with dog for at least 30 minutes per day; high being 5 or more days per week and low-medium being 4 or less days per week.

### Independent variables

The majority of items measuring the factors associated with dog walking behaviour were selected from previous surveys for consistency between studies [12–14, 22] and been tested for test-retest reliability with scores >0.7 [34]. Items were further supplemented based on additional findings from qualitative research with dog owners in the UK about potential motivators and outcomes perceived from dog walking [23, 37, 38].

Participant socio-demographic data collected included : number of people in the household; children <16 present in household; current age of participant; gender; highest education level; household income; dog ownership; marital status; job status; and, physical activity at work. Other owner-based questions included: self-rated general health; having a medical problem that prevents walking, height and weight (used to calculate BMI and categorise as normal, overweight or obese); and the validated Ten Item Personality Inventory (TIPI )[39].

Dog-related demographic factors collected and derived included [12]: number of dogs in the household; whether the participant only owned an old dog (10+ years); or only a small dog; whether at least one of the dogs was perceived to be overweight; or was a breed categorised by the UK Kennel Club as having high exercise needs (more than 2 hours a day); or owned a dog that attended a veterinary surgeon in the past year for a condition related to exercise.

Items measuring the dog-owner relationship and interaction [12] included: reasons for getting a dog; where the dog(s) slept; games played with the dog(s); whether the dogs lies on laps; whether any of the dog(s) bit or growled at household members of visitors; whether any dog(s) attended training classes; and dog attachment scale [40]. Attitudes and behavioural beliefs regarding dog walking included perceived health and behavioural benefits to dog, perceived health and social benefits to owner; perceptions of dog needs, and perceived behavioural control (PBC) [41], and perceptions of responsibility and obligation towards the dog. Other measures related to dog walking included normative beliefs and motivation to comply, self-efficacy [41], social support from family and friends for walking [33, 42] and an automaticity (habit) index adapted for dog walking [25].

### Ethical approval

The study protocol was approved by University of Liverpool Veterinary Research Ethics Committee (VREC334). Informed consent was obtained from all subjects. Households received an information flyer detailing the study at least a week before data collection began. Participants consented by completing and returning the questionnaires by post.

### Data analysis

Median frequency and durations of physical activity was compared between irregular and regular dog walkers using Kruskal-Wallis tests. Principal components analysis was used to create participant scores based on their common responses to questions about dog walking-related outcome evaluations, behavioural beliefs, perceived behavioural control, and attitudes. Principal component analysis was conducted using the ‘princomp’ function within the ‘factoextra’ library in R (https://cran.rproject.org/web/packages/factoextra/). Components were retained in order to retain a reasonable level of information whilst enabling sufficiently straightforward interpretability. We aimed to retain at least 60%-70% of the total variation by retaining components with the greatest (generally >1) eigenvalues.

Further statistical analyses were conducted in SPSS. Chi-squared tests and binary logistic regression analyses were conducted to test associations between variables of interest and the outcome of Regular Dog Walking. Due to the exploratory nature of this research, we used a stepwise regression approach to identify a subset of variables of likely primary importance. Initial selection of variables for inclusions in models incorporated a priori knowledge and theory. From among these variables, those with P<0.2 on univariable analysis as well as others considered to be likely to be associated with physical activity or to act as confounders of other relationships were taken forward to model building in blocks aligned to a socio-ecological approach to dog walking [11]. P<0.2 was chosen because it is fairly stringent within the pre-screening of 0.2, 0.25 or 0.3 often recommended [43, 44]. First a model of owner demographic factors (from Additional File Table 1) was built and backwards elimination used, with variables remaining in the model if they were significant (P<0.05) or if removal/addition resulted in substantial change to the effect of other variables. Owner gender, age, household income, married/partner and number of people in the household, were forced into the model despite being non-significant to adjust for basic demographic variables likely to be associated with physical activity in general. Second, a separate model was built for dog demographic, dog-owner interaction and reasons for getting a dog factors (from Additional File Tables 2, 3, 4) and the same process of backwards elimination was followed. Following, separate models were built incorporating: dog walking behaviour and beliefs (from Additional File Table 5): principal components variables (Additional File Table 6); social support factors (from Additional File Table 7); and normative beliefs (Additional File Table 8). Reports of social interactions that occurred during dog walking were found to be associated with dog walking, however it was inferred from previous research [23] that this was likely to be likely due to reverse causation (those who walk dogs more have more social interactions, but social interactions are not often a motivating factor to go dog walking) and therefore were not included as predictors in models here. Remaining factors were combined in a final model and any variables P>0.05 at this stage removed sequentially, to give the final model covering all domains. The fit of the final model was assessed using the Hosmer-Lemeshow statistic. As some respondents were grouped in households, the model was initially built accounting for this clustering using generalised linear mixed models including household as a random effect. However, inclusion of this random effect did not impact the outcomes and for simplicity was removed.

**Table 1:** Physical activity and dog walking (including jogging and cycling) in irregular and regular dog walkers

Characteristic	Irregular dog walkers (<150min/wk) (*n*=49)		Regular dog walkers (≥150 min/wk) (*n*=135)			
	Mean (SD)	Median (Quartiles)	Mean (SD)	Median (Quartiles)	*P* means	*P* medians
Minutes dog walking over a usual week	50.2 (46.0)	45.0 (0.0-78.8)	456.9 (306.5)	400.0 (240.0-600.0)	<0.001	<0.001
Frequency dog walking over a usual week	3.2 (4.2)	2.0 (0.0-4.0)	10.4 (6.0)	9.0 (7.0-14.0)	<0.001	<0.001
Minutes of total physical activity over a usual week	232.9 (232.8)	147.5 (71.3-322.5)	608.1 (343.6)	515.0 (367.5-810.0)	<0.001	<0.001
Achieve 150min physical activity over a usual week (%)	50%		100%			
Minutes of walking over a usual week	59.7 (58.6)	60.0 (30.0-120.0)	451.0 (307.1)	420.0 (250.0-630.0)	<0.001	<0.001
Minutes of walking for recreation over a usual week	51.8 (53.8)	30.0 (0.0-86.3)	422.3 (298.3)	360.0 (220.0-521.3)	<0.001	<0.001

**Table 2:** Principal components analysis of dog outcome evaluation

Dog Outcome Evaluation	Comp.1	Comp.2
Component name	Dog health and behaviour benefits	Dog health benefits
Component description	Benefits both health and behaviour	Benefits dog health but not behaviour
Outcomes:		
Benefits healthy weight maintenance	0.37	0.49
Improved quality of life	0.35	0.53
Decreased risk of disease	0.35	0.14
Decreased boredom by keeping an active body and mind	0.41	-0.09
Improved socialisation	0.39	-0.05
Fewer behaviour problems	0.39	-0.40
Decreased separation anxiety and less nervous when left alone	0.37	-0.55

Question - The benefits to dogs from being walked most days include

**Table 3:** Principal components analysis of behavioural belief strengths

Behavioural beliefs	Comp.1	Comp.2	Comp.3	Comp.4
Component name	Dog and owner benefits	Dog benefits	Concerns about other dogs	Owner physical benefits but difficult dog
Component description	Likely to impact owner physical and mental health and dog health and behaviour	Likely to benefit dog health and quality of life and unlikely to have problematic interactions with other dogs	Unlikely to promote positive interaction with dogs or people and fear of meeting out of control dogs	Likely to impact owner physical health but not benefit dog and dog might attack others
Beliefs:				
it would help me to do my own exercise	0.35	0.04	0.07	0.34
it would help me to lose weight	0.35	0.14	0.14	0.38
it would help me to relax	0.37	0.13	0.10	-0.10
it would be enjoyable	0.33	0.26	0.16	-0.16
it would allow me to get to know my neighbourhood	0.31	-0.09	-0.33	-0.16
it would allow me to get to know my neighbours	0.30	-0.17	-0.33	-0.40
it would stop me feeling guilty	0.20	-0.32	-0.41	0.22
it would reduce the risk of my dog(s) barking	0.24	-0.28	-0.26	-0.13
we would meet uncontrolled dogs that are off the lead	0.14	-0.44	0.40	-0.24
my dog might get bitten or attacked	0.11	-0.40	0.55	-0.20
my dog might attack other dogs or people	0.08	-0.41	0.06	0.59
it would improve my dog(s) quality of life	0.33	0.25	0.10	0.02
it would keep my dog healthy	0.27	0.29	0.12	-0.01

Question - Assuming you walked with your dog daily, how likely or unlikely is it that each of the following would occur

**Table 4:** Principal components analysis of perceived behavioural control (PBC)

PBC	Comp.1	Comp.2	Comp.3	Comp.4	Comp.5
Component name	Low PBC	Affected by dog factors and hygiene and unaffected by enjoyment	Easily affected by weather and health	Unaffected by working commitments and health	Affected by work and daylight
Component description	Agree that most aspects are likely to affect them to some agree	Likely to be affected by their own dog difficulties and by hygiene factors, but not their enjoyment of the outdoors, and interactions with other walkers or dogs	Strongly affected by weather and personal illness but not the environment or their own dog difficulties, or size of their garden	Strongly affected by shorter days and weather and hygiene factors, but not by day of week or working hours or family or health commitments	Affected by shorter days, day of week and working hours, but not environment or hygiene
PBC:					
The shorter days in winter	0.21	0.17	0.24	0.40	0.39
The weather eg (too hot, too cold, raining)	0.19	0.07	0.43	0.32	0.28
My family commitments	0.22	0.20	0.11	-0.15	0.01
If I was ill	0.20	0.03	0.40	-0.15	-0.05
If a family member was ill	0.21	0.02	0.24	-0.32	-0.19
The poor health or age of my dog	0.21	-0.04	0.29	-0.30	-0.30
Not having places to walk (e.g. parks)	0.22	0.17	-0.12	-0.22	-0.16
Dog owners not picking up after their dogs	0.23	0.29	-0.07	0.21	-0.34
The unavailability of dog poo bags	0.22	0.26	-0.18	0.15	-0.29
The lack of bins available for dog poo	0.24	0.29	-0.10	0.30	-0.21
Concern that my backyard/garden is too small	0.19	-0.05	-0.41	-0.18	0.16
The difficulty of walking two dogs	0.14	0.15	-0.29	0.06	0.07
My dog(s) would be unfriendly or difficult to control	0.21	0.19	-0.16	-0.04	0.11
My enjoyment of the outdoors	0.23	-0.24	0.04	0.00	0.03
The fact that I feel safe when walking my dogs	0.23	-0.28	-0.07	0.06	0.03
Seeing other people out walking	0.22	-0.36	-0.20	0.20	-0.03
Seeing other people out walking their dogs	0.22	-0.37	-0.13	0.21	-0.05
Knowing my dog enjoys going for a walk	0.24	-0.28	0.11	0.04	-0.04
Seeing my dog interacting with other dogs	0.26	-0.31	0.04	-0.03	-0.12
Day of the week (i.e. being a non-work day rather than work day)	0.22	0.05	-0.11	-0.34	0.38
My long working hours	0.26	0.15	-0.08	-0.22	0.40

Question - Assuming you tried to walk with your dog daily how likely are you to be affected by

**Table 5:** Principal components analysis of other attitudes to dog walking

Attitudes	Comp.1	Comp.2	Comp.3	Comp.4
Component name	High responsibility and enjoyment	Low responsibility and enjoyment despite dog need	Dog doesn’t need walks	Dog doesn’t like walks
Component description	Feels responsible/obligated, routine, dog and owner enjoyment, active breed	Lower responsibility and enjoyment but don’t perceive dog doesn’t need it or health as a barrier.	Feels responsible/obligated but dog is small/low activity breed and has company so needs less walks	Dog dislikes rain, is lazy and can exercise in garden
Attitudes:				
I feel an obligation to walk my dog daily	0.372411	0.073633	0.290149	0.112131
I feel a personal responsibility to walk my dog daily	0.381757	0.106079	0.32807	0.0736
Walking my dog(s) is part of my routine	0.386068	0.156494	0.192109	-0.0147
My dog(s) pressures me to take him/her for a walk daily	0.335032	0.044996	0.046263	0.158412
My dogs are at their happiest on a walk	0.368659	-0.04921	-0.14688	-0.11596
I feel happy when I see my dogs acting happy on a walk	0.388985	-0.02056	-0.19699	-0.21415
My breed of dog(s) requires a lot of exercise	0.239218	0.148158	-0.28372	0.19913
It feels wrong to go for a walk without my dog(s)	0.27376	-0.03729	-0.34853	-0.22248
My health prevents me walking my dog as much as I would like	0.054359	-0.36157	0.171443	-0.21292
My dog(s) have each other for exercise and company so need walking less	0.021239	-0.36471	0.273883	-0.13336
My dog(s) are small so do not require as much exercise as bigger dogs	0.027529	-0.35608	0.39083	-0.39875
My dog does not like the rain/cold	0.148586	-0.3928	-0.11741	0.248215
My dog(s) gets its exercise in the garden	0.009171	-0.40543	-0.03194	0.423812
My dog is lazy	0.070318	-0.39213	-0.12697	0.433722
As long as someone walks the dog it doesn’t have to be me	0.060151	-0.2601	-0.46766	-0.39595

Question - To what extent do you agree or disagree with the following statements

**Table 6:** Multivariable model of reporting 150mins of walking with their dog per week (regular dog walking)

Variable	Level	OR	95%CI	P
Number of people in household	1	1		0.08
	2	0.03	0.001-0.79	0.04
	3+	0.07	0.003-1.62	0.10
Owner gender	Male	1		
	Female	1.35	0.44-4.13	0.59
Owner age (years, categorised)	16-29	1		0.33
	30-49	0.20	0.21-1.87	0.16
	50-69	0.50	0.06-4.16	0.52
	70+	1.02	0.07-15.03	0.99
Marital/partner status	Unmarried/does not live with partner	1		
	Married or lives with partner	33.51	2.48-458.84	**0.01**
Household income	£0-20,000	1		0.26
	£20-40,000	4.59	0.83-25.51	0.08
	£40-60,000	1.32	0.25-6.99	0.75
	£60,000+	2.40	-.38-15.27	0.35
Intention in next month to walk with dog for at least 30 minutes per day	Low-medium (4 days per week or less)	1		
	High (5 days per week or more)	12.09	3.45-42.32	**<0.001**
Dog walking automaticity (habit) index	Continuous score (4-20)	2.09	1.25-3.51	**<0.01**
Friends provided social support for walking in past month	Average score 1-5	0.27	0.11-0.68	**<0.01**

Hosmer-Lemeshow Statistic 0.66. *n*=130. Bold=*P*<0.05

**Table 7:** Multivariable model of high intention to walk the dog

Variable	Level	OR	95%CI	P
Number of people in household	1	1		0.15
	2	0.01	0.00-1.94	0.09
	3+	0.01	0.00-1.21	0.06
Owner gender	Male	1		
	Female	4.67	1.18-18.53	**0.03**
Marital/partner status	Unmarried/does not live with partner	1		
	Married or lives with partner	12.84	0.93-176.79	0.06
Household income	£0-20,000	1		0.33
	£20-40,000	0.70	0.07-6.76	0.76
	£40-60,000	0.30	0.04-2.24	0.24
	£60,000+	0.19	0.02-1.68	0.14
Dogs lie on sofa	Never/rarely	1		
	Sometimes/often	6.85	1.48-31.76	**0.01**
Someone else walks dog	Nobody else walks dog	1		
	Someone else walks dog (eg spouse, child, family member, friend, other)	0.09	0.01-0.69	**0.02**
Dog walking Attitudes component	High responsibility and enjoyment	2.10	1.16-3.82	**0.02**
Self efficacy to walk with dog most days next month	Low-moderate	1		
	High	5.82	1.54-21.94	**0.01**
Dog walking automaticity (habit) index		1.94	1.02-3.72	**0.05**

Hosmer-Lemeshow Statistic 0.95. *n*=127. Bold=*P*<0.05

A similar process of model-building was also applied to the outcome of high intention to dog walk. Here, owner age could not be included in the model due to model stability as it resulted in unusually high figures and wide confidence intervals. The belief variables ‘Having a dog makes me walk more’ and ‘In the past month my dog encouraged me to walk’ were not included in model building again due to theoretical reverse causation in that owners who regularly walked their dogs in the past likely had high intention to walk their dogs in the future also.

## Results

### Descriptive analysis

Activity data were skewed so medians are presented, however means are also presented in Table 1 for comparison, because previous physical activity and dog walking studies have often reported means, (for example [14]). Almost all of dog-related physical activity was from walking only (median 7.0 times per week; median 220.0 mins per week). Within those who walked with their dog, it occurred for a median 7.0 times and median 247.5 mins per week. Only 5.3% of owners jogged with their dogs (for a median 2 times a week, 60 mins per week) and 2.1% cycled (for a median 2 times a week, 180 mins per week). A small number of people reported doing agility training with their dogs (2.1%) and other forms of training (obedience, rally, gundog) (3%). Combined ‘dog walking’ (excluding agility/training but including jogging and cycling) occurred a median 7.0 times per week and for 230 mins per week.

Sixty-five percent of dog owners met the criteria of regular dog walkers (at least 150mins/week). Regular dog walkers walked with their dog a median of 400 mins/week, whereas irregular dog walkers walked with their dog a median of 45 mins per week (P<0.001) (Table 1). Regular dog walkers walked with their dog a median 9 times per week whereas irregular dog walkers walked with their dog a median 2 times per week (P<0.001). On average, walking with a dog contributed to a median 81% of a regular dog walker’s total physical activity and median 100% of their weekly minutes of walking. For irregular dog walkers the contribution of dog walking to weekly minutes of total physical activity was less than a third (median 26%) than that of regular dog walkers (P<0.001). The contribution to total walking was also slightly lower (median 95%) (*P*=0.01).

### Principal components analysis

**Fig. 1:**
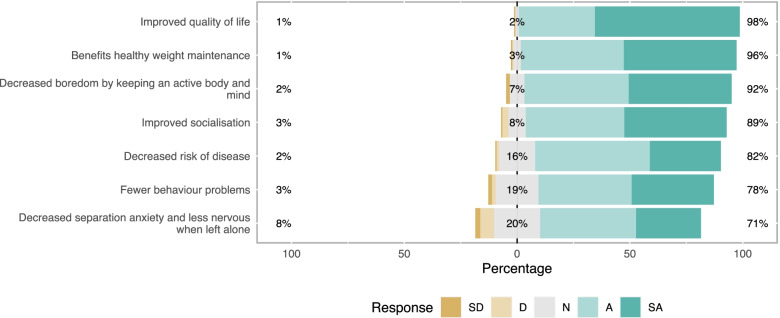
Dog outcome evaluation survey responses of 191 dog-owning adults in a community in Cheshire, UK. Question - The benefits to dogs from being walked most days include (strongly disagree; disagree; neither agree nor disagree; agree; strongly agree)

**Fig. 2:**
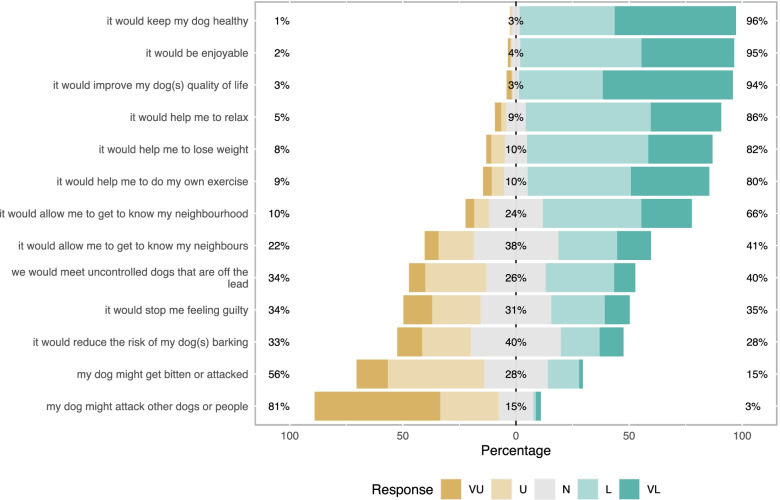
Behavioural belief strength survey responses of 191 dog-owning adults in a community in Cheshire, UK. Question - Assuming you walked with your dog daily, how likely or unlikely is it that each of the following would occur (very unlikely; unlikely; neither unlikely or likely; likely; very likely)

**Fig. 3:**
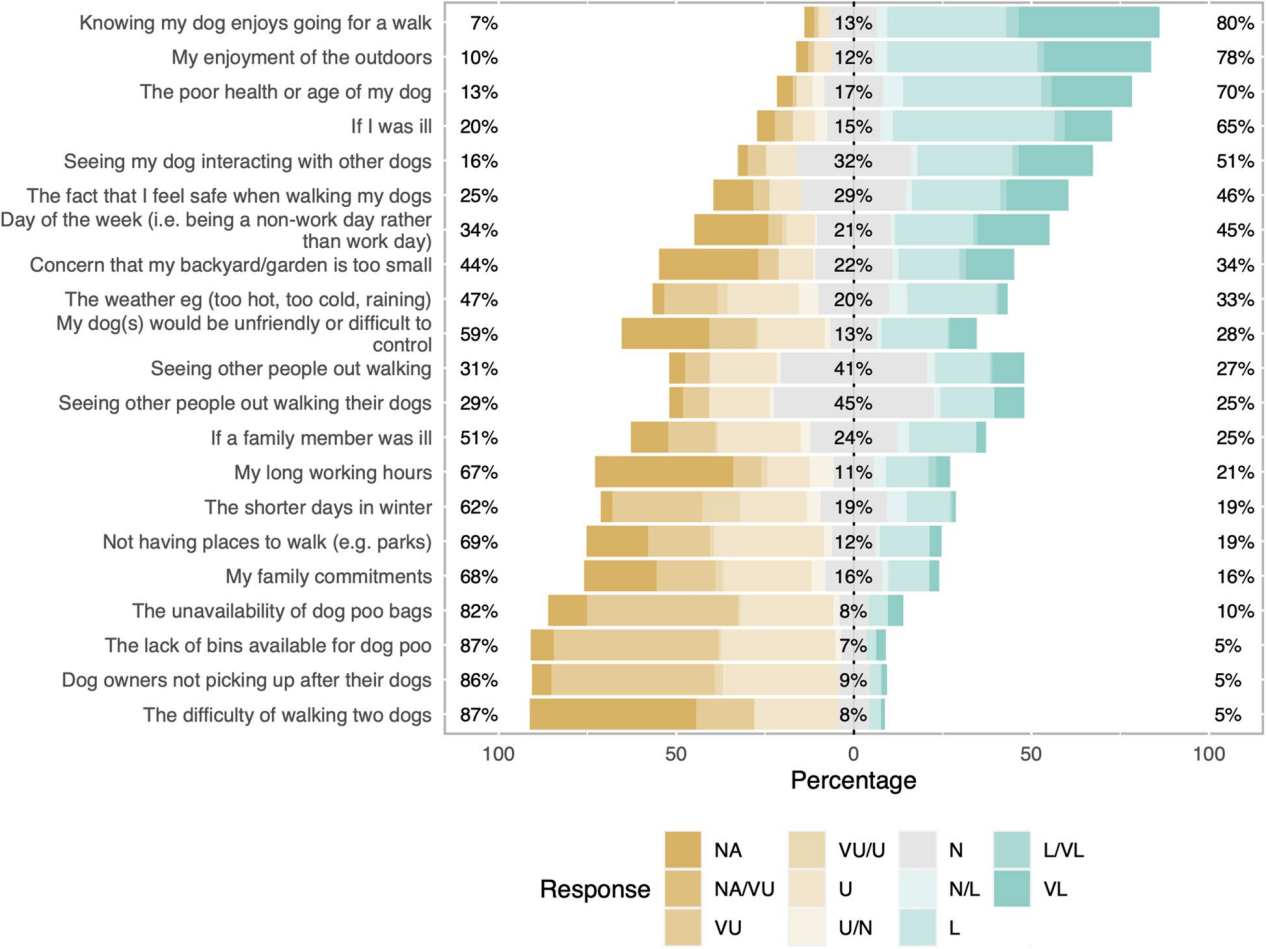
Perceived behavioural control (PBC) survey responses of 191 dog-owning adults in a community in Cheshire, UK. Question - Assuming you tried to walk with your dog daily how likely are you to be affected by (very unlikely; unlikely; neither unlikely or likely; likely; very likely). Scores averaged from responses to two options – 1) would make me walk my dog less often, 2) would make dog walks shorter

**Fig. 4:**
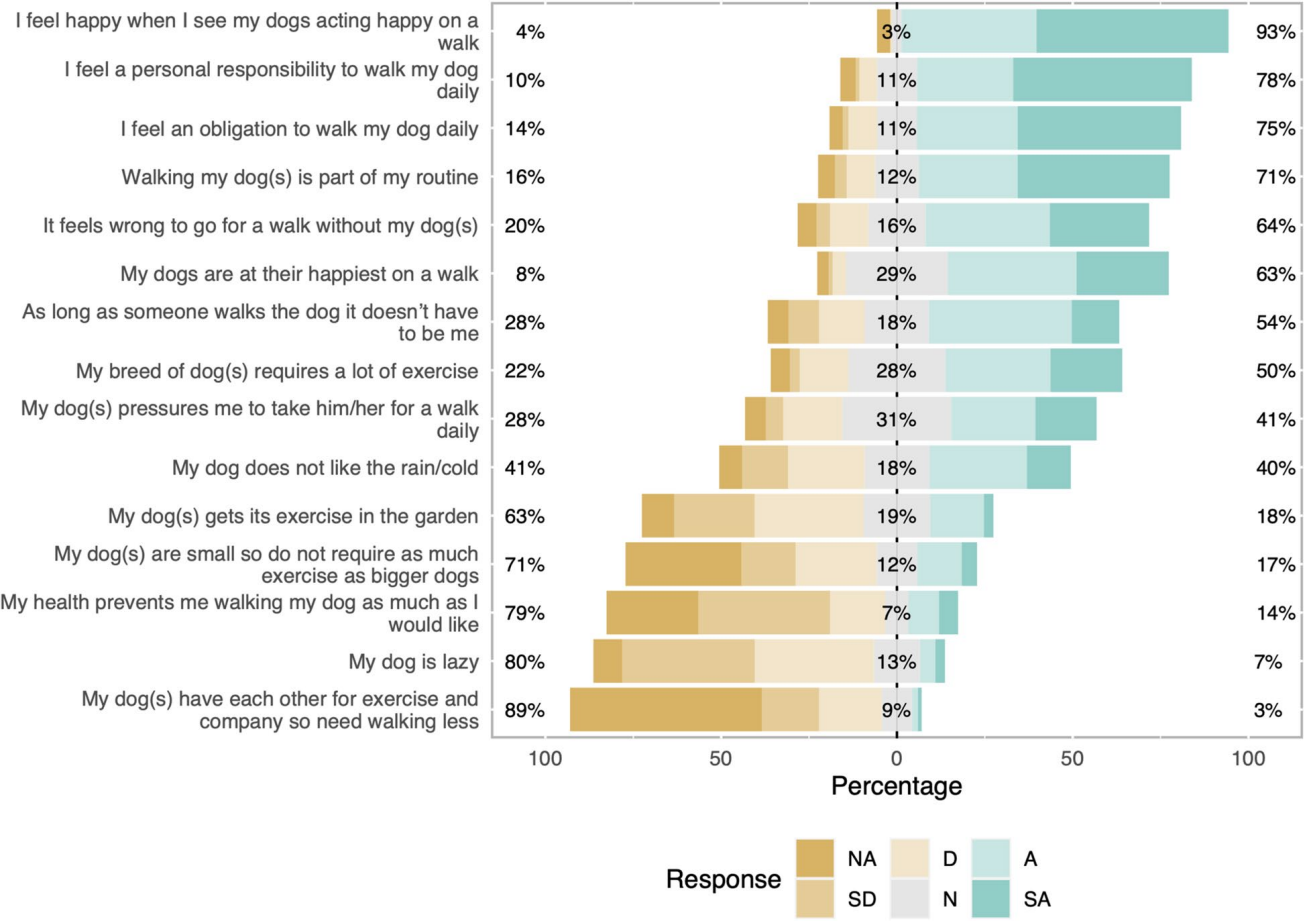
Other attitudes to dog walking survey responses of 191 dog-owning adults in a community in Cheshire, UK. Question - To what extent do you agree or disagree with the following statements (not applicable; strongly disagree; disagree; neither agree nor disagree; agree; strongly agree)

Responses to questions relating to attitudes and behavioural beliefs about dog walking are presented in Figs. 1, 2, 3, 4. Principal components analysis (Tables 2, 3, 4, 5) identified two types of owner attitudes to dog walking related to perceived dog outcomes (behaviour and health; health only); four behavioural beliefs (dog and owner benefits; dog benefits; concerns about other dogs; owner physical benefits but difficult dog); five perceived behavioural controls (Generally low perceived behavioural control; affected by dog factors and hygiene and unaffected by enjoyment; easily affected by weather and health; unaffected by working commitments and health; affected by work and daylight); and four other attitudes to dog walking (high responsibility and enjoyment; low responsibility and enjoyment despite dog need; dog doesn’t need walks; dog doesn’t like walks).

### Univariable analysis

Univariable findings for both regular dog walking and high intention to dog walk are presented in Additional File 1.

### Multivariable analysis – Regular dog walking

The final multivariable model is presented in Table 6. Participants with a high intention to walk their dog in the next month had higher odds of being a regular dog walker (OR=12.09, 95%CI=3.45-42.42, *P*<0.001), compared with participants with low/median intention to walk their dog. Regular dog walkers also scored significantly higher on the habit/automaticity index for dog walking (OR=2.09, 95%CI=1.25-3.51, *P*<0.01) but had lower odds of high support from friends for walking (OR=0.27, 95%CI=0.11-0.68, *P*<0.01). Being married or living with a partner was associated with higher odds of regular dog walking (OR=33.51, 95%CI=2.48-458.84, *P*=0.01), although the wide confidence intervals mean this should be interpreted with caution.

### Multivariable analysis – High intention to walk

Female owners had higher odds of high intention to dog walk in the next month compared to male owners (OR=4.67, 95%CI=1.18-18.53, *P*=0.03; Table 7). Owners whose dogs sometimes or often laid on the sofa were more likely to have a high intention to dog walk than dogs who never or rarely did this (OR=6.85, 95%CI=1.48-31.76, *P*=0.01). Owners who reported attitudes of feeling a responsibility to walk their dog and enjoyment of dog walking were more likely to have a high intention to dog walk (OR=2.10, 95%CI=1.16-3.82, *P*=0.02). Similarly, those reporting high feelings of self-efficacy to walk their dog most days had higher odds of a high intention to dog walk compared to those with low-moderate self-efficacy (OR=5.82, 95%CI=1.54-21.94, *P*=0.01). Those with a high intention to dog walk also had higher scores on a dog walking habit index (OR=1.94, 95%CI=1.02-3.72, *P*=0.05). Finally, reporting someone else walking the dog (as well as, or exclusively) was associated with a lower odds of high intention to dog walk (OR=0.09, 95%CI=0.01-0.69, *P*=0.02), even after adjustment for number of people in the household.

## Discussion

This novel study examined factors associated with owners in a UK population walking their dog for 150 minutes per week or more (which we termed regular dog walking), and, also for the first time, factors associated with having a high intention to dog walk. Having a high level of intention to walk the dog in the next month (5 days a week or more) was found to be positively associated with regular dog walking compared to medium/low intention (4 days a week or less). In addition, other factors positively associated with regular dog walking were being married/living with a partner compared to not (after already accounting for number of people in the household), and higher scores on a dog walking habit index. However, higher support from friends for walking was found to be associated with lower odds of regular dog walking. High intention to dog walk was associated with female owners compared to male, dogs lying on the sofa sometimes or often compared to rarely or never, high levels of perceived self-efficacy to walk the dog over the next month compared to low-moderate, the attitude of high responsibility and enjoyment from dog walking, and higher scores on a dog walking habit index. Those who reported someone else in the household also walks the dog had a lower odds of high intention to dog walk compared to participants who did not report someone else walking the dog.

This study also outlines a number of owner profiles based on attitudes and beliefs about dog walking, that echo factors identified in previous qualitative or quantitative studies, and demonstrates more explicitly how these attitudes group together. Some owners emphasised that walking the dog was beneficial to the dog’s physical health, whereas others felt it was beneficial for both dog health and behaviour [22]. Some owners felt that dog walking was beneficial mainly to their dog, whereas others felt that it was beneficial to both the owner and the dog [23]. One owner type believed that there were physical benefits to the owner from walking, but their dog was unlikely to benefit and dog walking was made difficult by their dog’s behaviour such as attacking other dogs [23, 38]. Some owners reported considerable concerns about the behaviour and impact of other dogs that they meet when out walking [23, 45]. Whilst there were owners that felt a high level of responsibility to walk their dog and enjoyment of dog walking, others felt a low level of responsibility and enjoyment despite feeling that their dog needs walks (perhaps because the dog was walked by other household members instead of them) [23, 37]. Some owners felt that their dog did not like bad weather and is lazy [23] and can exercise in the garden [45]. Another element was the owner perception that their dog is small or a low activity breed [22, 23, 45] and has company so doesn’t require much walking, however interestingly whilst still reporting feeling a responsibility to walk their dogs. Some owners felt low perceived behavioural control over dog walking and were likely to be impacted by many factors preventing them. Others were likely to be affected by dog-related difficulties, some by the weather and their own health issues, others by the weather but not their work commitments or health, and others by both the weather and their work commitments [23]. These findings elucidate that owner attitudes and approaches to dog walking, including some previously identified in other studies, actually group into a number of different and distinct dog-walker profiles.. These can now be used to inform the design of potential interventions to promote more dog walking and increase physical activity levels, so that they can be better targeted to owner types and have potential increased efficacy.

Perhaps unsurprisingly, our findings show that in this UK sample people were more likely to walk their dog if they had formed an intention to do it. This may be in part due to reverse causality; if you previously walked your dog you are probably more likely to intend to do it in the future. However, intention is known to predict a considerable amount of physical activity variability, although it does not explain all physical activity undertaken, known as the intention-behaviour gap; action control describes people’s ability to translate intention into action [46]. Therefore, our study elucidates that it is important that intention to dog walk is considered in terms of how it can be used to motivate people to dog walk, but there will still be further barriers to walking to address, despite this good intention. We also found that automaticity/habit formation was associated with regular dog walking (whilst controlling for intention to dog walk), and also associated with having the intention to walk the dog, as hypothesised. This supports previous research suggesting that creating habits and routines around dog walking is important for maintaining behaviour [23, 25].

The outcomes identified through principal components analysis created new predictor variables and the finding that the type of owners who scored highly on the component of enjoyment of walking and feelings of responsibility to walk the dog were more likely to have a high intention to walk the dog. This quantitatively supports our qualitative research which identified that dog walking is viewed by owners as an enjoyable means of stress relief and relaxation [23, 38], and as an important part of the responsibility of dog ownership in order to do what is morally best for the dog [37]. Those with a high intention to walk the dog were also more likely to score high on feelings of self-efficacy to walk the dog, which fits with the previous observation that self-efficacy predicts stages of change for dog walking [19].

Our study suggests that a barrier to having a high intention to walk the dog is if someone else in the house walks the dog, which is similar to our previous study that also suggested that having a spouse/partner/child who walks the dog is negatively associated with a different outcome of feeling motivation and encouragement to dog walk [22]. However, in contrast we also found that being married/living with a partner is positively associated with actual regular dog walking (after adjustment for number of people in the household) and is in support of findings from a Japanese study of older adults [18]. This may be because dog walking can be an activity people enjoy doing together with a close family member, as found in qualitative studies [38]. In contrast, in other research cardiovascular benefits were only seen in single-person dog-owning households (potentially explained where responsibility for dog walking is likely more difficult to pass to another person) [47]. However, we did not find that that having someone else who walked the dog, was associated with actual walking behaviour (only intention), in agreement with other studies that found that walking behaviour did not appear to be associated with a spouse/other family member walking the dog [13, 14, 21] A suggested explanation for this complexity is that perhaps another person being involved in dog walking is not a clear barrier (as multiple people can walk dogs together), but the perception that someone else can/will do it instead may be problematic for forming an intention to walk in the future. The type of relationship may also matter; dog walking together with a partner may be a particularly supportive and enjoyable experience that household members can do together, compared to how dog walking duties may be delegated when with living in other types of shared accommodation. Thus, interventions to increase dog walking participation may need to address ways to encourage multiple household members to be involved in the responsibility for walking the dog, either through increased number and duration of walks for the dog, or through multiple people co-walking together. The barriers for this are likely to be situation specific, but previous research suggests they may involve a perception of time pressures and how to allocate competing daily tasks [23, 37, 38]. Creating a household level routine or habit, of who walks the dog when, may be helpful.

This study also suggests that regular dog walkers were less likely to feel supported by friends in walking. Although this may not sound intuitive, it may be because regular dog walking behaviour is already a strong habit in regular dog walkers, and thus they are in the maintenance phase behaviour change rather than contemplation or action phases [48] which might be expected to be influenced by support and motivation from friends for walking. It may also be explained by the nature of dog walking being primarily a solitary or household activity rather than something often done with friends.

Another unexpected finding was our observation that female owners had a higher intention to dog walk, but were not more likely to be regular walkers once intention to walk the dog was adjusted for. Previous research into the factors associated with feeling encouraged and motivated by the dog to walk has not reported gender differences [22] nor were there these seen when investigating types of dog walkers regarding their intention and action control [25]. Our finding may be due to unaccounted confounding but this is probably unlikely due to the large effect size found. Although women are generally known to be slightly less physically active than men [3], they are historically placed as the main caregivers including of domestic dogs [49] and may also be more likely than men to perform recreational walking [50] which may explain their higher intention to walk dogs in our study.

In addition, owning dogs who were reportedly allowed to lie on the sofa more often was associated with a high intention to dog walk. This may reflect differences in qualities of the relationship between the owner and the dog, perhaps indicating a stronger or different quality of bond, however, our measure of owner attachment to the dog did not appear to be associated with intention to dog walk nor regular dog walking. It has previously been shown that small dogs are more likely to be are allowed on the sofa rather than larger [51] but given that studies have reported that larger dog size is associated with dog walking [12, 52], and our study provides further evidence to those showing no association [13, 18] (as we found no evidence that dog size nor being a high exercise breed was associated with either walking behaviour or intention to walk whilst accounting for other factors), it is unlikely that dog size is confounding the sofa-walking potential relationship. . These areas require further investigation.

Our study has a number of strengths and limitations. It is the first study in the UK to investigate factors associated with owner participation in dog walking and the first in the world to study factors associated with having the intention to walk the dog. It also collected information from multiple household members living with the dog rather than a single dog owner as in previous studies (likely the person who walks the dog the most), whilst effectively controlling for access to suitable walking locations, which is difficult to measure in previous research, by this time recruiting households from the same geographic neighbourhood. However, the sample size was small which is reflected in wide confidence intervals, suspiciously large effect size estimates in some cases, and likely low power to detect some effects or erroneous detection of others. Therefore, interpretations and applications of the findings should be made with caution, as they may not be accurate, and in particular, we have mainly refrained from referring to effect sizes in the discussion. In particular for this reason we were unable to model the impact of owner age on intention to dog walk, and this should be investigated in future studies. The study was also survey-based measuring self-reported rather than objective dog walking behaviours, which would obviously be more preferable.

## Conclusions

This novel study suggests that an important consideration for encouraging higher human physical activity levels through dog walking, and improved canine welfare, is the need to motivate intention to walk the dog, particularly in male owners, who had a lower intention to walk than female owners. Intention may be motivated through increasing feelings of responsibility to walk the dog, increasing owner enjoyment of dog walking, and establishing habits/routines around dog walking, However, whilst owners’ motivation and intention to walk the dog are key factors, it is important to understand that this alone will not result in regular dog walking behaviour. Hence, interventions to facilitate the translation of intention into action are also required – these need to address issues of self-efficacy and how to overcome challenges and interruptions to plans, in particular as perception of self-efficacy was associated with intention to dog walk. Finally, our study demonstrates that interventions to promote dog walking also need to address attitudes around who walks the dog, in particular whether this responsibility can be shared and made a habit/routine that multiple household members can benefit from.

## Data Availability

The datasets used during the current study are available from the corresponding author on reasonable request.

## Abbreviations

RDW: Regular dog walker (≥150 min/wk)
IDW: Irregular dog walker <150 min/wk)
PBC: Perceived behavioural control
TIPI: Ten-item personality inventory
DAPA: Dogs and physical activity tool
NPAQ: Neighbourhood physical activity questionnaire
